# Oxidative Stress Inducers in Cancer Therapy: Preclinical and Clinical Evidence

**DOI:** 10.3390/antiox12061159

**Published:** 2023-05-26

**Authors:** Zohra Nausheen Nizami, Hanan E. Aburawi, Abdelhabib Semlali, Khalid Muhammad, Rabah Iratni

**Affiliations:** 1 Department of Biology, College of Science, United Arab Emirates University, Al Ain PO Box 15551, United Arab Emirates; 202170108@uaeu.ac.ae (Z.N.N.); 700039455@uaeu.ac.ae (H.E.A.); k.muhammad@uaeu.ac.ae (K.M.); 2 Groupe de Recherche en Écologie Buccale, Faculté de Médecine Dentaire-Université Laval, Quebec, QC G1V 0A6, Canada; abdelhabib.semlali.1@ulaval.ca

**Keywords:** reactive oxygen species, cancer therapy, phytochemicals, oxidative stress

## Abstract

Reactive oxygen species (ROS) are metabolic byproducts that regulate various cellular processes. However, at high levels, ROS induce oxidative stress, which in turn can trigger cell death. Cancer cells alter the redox homeostasis to facilitate protumorigenic processes; however, this leaves them vulnerable to further increases in ROS levels. This paradox has been exploited as a cancer therapeutic strategy with the use of pro-oxidative drugs. Many chemotherapeutic drugs presently in clinical use, such as cisplatin and doxorubicin, induce ROS as one of their mechanisms of action. Further, various drugs, including phytochemicals and small molecules, that are presently being investigated in preclinical and clinical studies attribute their anticancer activity to ROS induction. Consistently, this review aims to highlight selected pro-oxidative drugs whose anticancer potential has been characterized with specific focus on phytochemicals, mechanisms of ROS induction, and anticancer effects downstream of ROS induction.

## 1. Introduction

Reactive oxygen species (ROS) are molecules that contain one or more unpaired electrons, which contribute to their high reactivity. ROS can be classified as free radicals and nonradical molecules [1]. Examples of free radicals include superoxide (O_2_^•−^), hydroxyl (HO^•^), and peroxyl (RO_2_^•−^) radicals, and nonradical molecules include hydrogen peroxide (H_2_O_2_) and organic peroxides (ROOH). Physiologically, ROS are produced as byproducts of metabolic reactions, namely electron leakage during oxidative phosphorylation in the mitochondria, through the activity of NADPH oxidases, and via the iron-dependent Fenton reaction [2,3]. In normal cells, intracellular ROS levels are regulated by antioxidants and maintained at levels that regulate various signaling pathways, including cell proliferation, metabolism, and differentiation, among others. At levels beyond the physiological threshold, ROS cause oxidative damage to various cellular components, namely nucleic acids, proteins, and lipids, which in turn can induce cell death [4].

In cancer cells, the redox balance is disrupted due to several factors, including activation of oncogenes, aerobic glycolysis, and hypoxia. This can result in the accumulation of ROS, which can be lethal to cancer cells [3,5]. Therefore, to combat this issue and prevent oxidative stress-induced cell death, cancer cells employ several mechanisms, such as enhanced expression and activity of components of the antioxidant defense system, for example, peroxide scavenging systems that reduce H_2_O_2_ to H_2_O. Additionally, the availability of O_2_, mitochondrial localization in the cell, and the rates and concentrations of electrons and their carriers in the electron transport chain further regulate the production of ROS, specifically mitochondrial ROS [6]. Collectively, this maintains ROS at levels that facilitate activation of protumorigenic signaling pathways, thereby driving tumor progression, without inducing oxidative stress-induced cell death. For example, ROS can affect mRNA regulation and increase the expression of G1/S cyclins, thereby promoting cell cycle progression in breast cancer through the ERK1/2 MAPK pathway [7]. Similarly, ROS were found to be integral for anchorage-independent growth induced by the protooncogene *KRAS* [8]. In addition, mitochondrial ROS are known to act as a stabilizer of hypoxia-inducible transcription factor-1α, which regulates the expression of vascular endothelial growth factor, which in turn activates pathways that lead to the proliferation of endothelial cells, ultimately promoting angiogenesis [9]. Similarly, other signaling pathways can also be activated by ROS in cancer cells to drive their progression, given their role as intracellular messengers. 

**Figure 1:**
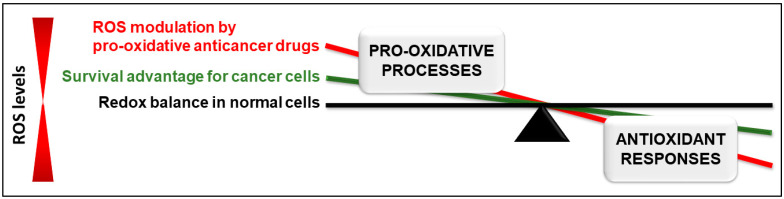
The paradoxical role of reactive oxygen species (ROS) in cancer. In normal cells, ROS levels are tightly regulated by a balance between pro-oxidative cellular processes and the antioxidant responses. Several factors can increase ROS production in cancer cells to levels which provide a survival advantage by activating tumorigenic pathways to drive tumor progression. This can be therapeutically exploited through ROS modulation with pro-oxidative drugs which tip the already skewed redox balance and further increase ROS production to levels that mediate oxidative stress-induced cell death. ROS—reactive oxygen species.

However, despite the survival advantage, the altered redox homeostasis increases the susceptibility of cancer cells to further changes in ROS levels, and therefore increases their susceptibility to oxidative stress-induced cell death. Hence, pro-oxidative drugs which skew this altered redox balance to further increase ROS levels have been proposed and investigated as a therapeutic strategy for cancers [2,3,5,10] (Figure 1). Induction of ROS by pro-oxidative drugs can be modulated through different mechanisms. It can be attributed to: (1) direct ROS induction as a consequence of drug metabolism (e.g., doxorubicin, which is metabolized to its unstable semiquinone radical); (2) upregulation of pro-oxidative enzymes, such as NADPH oxidase, which produce superoxide; or (3) targeted inhibition of cellular antioxidant mechanisms, such as superoxide dismutase (SOD) 1, which catalyzes the conversion of oxide ion (O_2_^−^) produced by the electron transport chain or as byproducts of other enzymatic reactions into H_2_O_2_ [11]. 

Indeed, many chemotherapeutic drugs exhibit their anticancer effects through direct or indirect induction of oxidative stress. This review aims to highlight anticancer drugs that attribute their anticancer activity primarily to ROS induction, such that the use of ROS inhibitors/scavengers abrogates their anticancer effects. In addition to the above-mentioned rationale, we further narrowed down the drugs highlighted in this review to meet the following criteria: (1) they exhibit their anticancer activity in two or more cancer types; (2) the pathways that modulate their anticancer effects are induced downstream of ROS. Moreover, given the growing interest in phytochemical compounds as anticancer therapeutics, we specifically focused on pro-oxidative phytochemicals and further classified them based on whether the mechanism of ROS induction has been elucidated.

## 2. Pro-Oxidative Drugs in Preclinical Study

This section discusses pro-oxidative anticancer drugs, with special emphasis on phytochemicals, which are currently being investigated in preclinical studies. 

### 2.1. Phytochemicals with a Characterized Mechanism of of ROS Induction

An overwhelming proportion of anticancer drugs that have been approved in the past decades are derivatives of natural products. For example, paclitaxel, a taxane diterpene that is widely used for the treatment of various cancers, including breast, endometrial, and ovarian cancers, was originally derived from the Pacific yew (*Taxus brevifolia*) [12]. 

This section highlights selected pro-oxidative phytochemical compounds that attribute their anticancer activity to ROS-mediated mechanisms, and whose mechanism of ROS induction has been characterized. 

#### 2.1.1. Piperlongumine

Piperlongumine (PPL) is an alkaloid/amide that was identified in root extracts of long pepper (*Piper longum*) and has been investigated for its anticancer activity in various cancer types, including hematological cancers, colorectal, gastric, lung, breast, prostate, and oral cancers, melanoma, and glioma [13,14]. Its in vitro anticancer activity can be attributed to induction of ROS through increased glutathione disulfide levels, decreased glutathione levels, and inhibition of thioredoxin reductase (TrxR), an enzyme which reduces thioredoxin, a redox protein that protects against oxidative stress [13,14]. PPL-mediated ROS accumulation further leads to ROS-mediated apoptosis [15], G1 or G2/M cell cycle arrest [16,17], ER stress [15], and oxidative DNA damage [17]. PPL was also found to modulate various signaling pathways involved in tumor progression, including the JAK/STAT3, ERK, NF-κB, and PI3K/AKT/mTOR pathways [13,14]. Consistent with the regulation of these important pathways, which are also involved in chemoresistance, PPL was reported to sensitize head and neck, gastric, and liver cancers to cisplatin [18], oxaliplatin [19], and sorafenib [20], respectively, through induction of ROS both in vitro and in vivo. However, despite the promising potential of PPL as both an anticancer therapeutic and a chemosensitizing agent, its pharmacokinetics have not been clarified. Additionally, its poor aqueous solubility and bioavailability limit its therapeutic potential [13,14]; however, efforts have been made on this front in relation to drug delivery systems, especially with nanoparticles and nanoemulsions, which have shown promising results [14,21,22]. 

#### 2.1.2. Resveratrol

Resveratrol (3,5,4′-trihydroxy-trans-stilbene) is a stilbenoid that is found in various plant species, including common grape vine (*Vitis vinifera*) and berries of the genus *Vaccinium*, and has been investigated for its biological activities including anticancer, anti-inflammatory, and antimicrobial properties, among others. The anticancer activity of resveratrol is well characterized both in vitro and in vivo in various cancer types [23]. Several molecular mechanisms have been proposed for the anticancer activity of resveratrol, including ROS induction. However, with respect to ROS, the effect of resveratrol appears to be concentration dependent; at low concentrations, it exerts antioxidant effects, whereas at high concentrations (50–100 µM), resveratrol induces ROS production [24,25], which can be attributed to increased NADPH oxidase activity [25]. Resveratrol-induced ROS accumulation modulates autophagy and apoptosis in colon [25,26], pancreatic [27], and bladder cancer [28] cells. Cheng et al. [27] reported that resveratrol-induced ROS activate the Nrf2 signaling pathway, which subsequently suppresses NAF1 and induces apoptosis in pancreatic cancer cells. This also increased their sensitivity to gemcitabine. Additionally, resveratrol induces ROS-mediated DNA damage [24,29], which has been reported to mediate senescence through the DLC1–DYRK1A–EGFR axis in breast and liver cancer in vitro and in vivo [29]. Despite the promising potential of resveratrol, its unstable pharmacokinetics due to its high metabolism and poor bioavailability limit its clinical application. Consistently, resveratrol analogues, such as 3,4,4′-trihydroxy-trans-stilbene (a synthetic analogue) [30] and piceatannol (a natural analogue) [31], have been investigated to overcome the same and are more potent, bypassing the high dosage issue, and were also found to exert their anticancer activity through ROS induction. Moreover, various nanocarriers have been investigated to optimize drug delivery and improve bioavailability; although many have been studied in vitro, in vivo studies are lacking for the same and are hence warranted [32].

#### 2.1.3. Oleanolic Acid 

Oleanolic acid (OA, 3β-hydroxyolean-12-en-28-oic acid), a pentacyclic triterpenoid, is found in various plant extracts and is one of the bioactive components of ginseng (*Panax* spp.) and olive (*Olea europaea*). OA and its derivatives have been studied extensively for their various biological activities, including anticancer activity, which has been characterized in vitro and in vivo in various cancers including hepatocellular, breast, colon, prostate, melanoma, and hematological cancers [33]. ROS induction and accumulation by OA has been reported in lung, pancreatic, osteosarcoma, and prostate cancer cells [34,35]. Consistently, its derivatives SZC017 [36], per-O-methylated-β-cyclodextrin-conjugated oleanolic acid [37], methyl-2-cyano-3,12-dioxooleana-1,9(11)-dien-28-oate [38], diclofenac-oleanolic acid oxime derivative conjugates [39], and olean-28,13b-olide 2, and a gold(I) complex containing an oleanolic acid derivative (4 b) [40], also increase ROS levels; the latter three induce ROS by downregulating the expression of glutamine transporter SLC1A5, antioxidant enzymes SOD1 and NAD(P)H dehydrogenase [quinone] 1, and TrxR, respectively. OA-mediated ROS accumulation induces cell cycle arrest (G0/G1 and G2/M) [34,36,41], autophagy [36], ER stress [40], mitochondrial membrane depolarization [37,42], and mitochondria-dependent apoptosis [37,42] through modulation of various signaling pathways. The following signaling pathways were reported to be modulated by OA and/or its derivatives in an ROS-dependent manner: activation of p38/MAPK pathway in lung, pancreatic, and osteosarcoma cells [35]; inhibition of PI3K/AKT pathway in gastric, prostate, and lung adenocarcinoma cancer cells [34,36,43]; and inhibition of NFkB pathway in lung adenocarcinoma and hepatoma cells [41,43]. Moreover, OA and its derivatives also function as chemosensitizers and have been reported to increase the sensitivity of hepatocellular cancer cells to sorafenib [42,44] and lung adenocarcinoma cells to cisplatin [43]. Collectively, these studies highlight the promising potential of OA and its derivatives as anticancer agents. However, like other phytochemical compounds, its clinical use is limited by poor bioavailability, and although several derivatives have improved its bioavailability, further research is still needed on this front.

#### 2.1.4. Plumbagin

Plumbagin (5-hydroxy-2-methyl-1,4-napthoquinone) is a naphthoquinone found in the roots of Leadwort (*Plumbago zeylanica* L.), and its anticancer activity has been well characterized against various cancers, including breast cancer, melanoma, glioma, hepatocellular cancer, oral squamous cell cancer, and T-cell lymphoma, among others [45]. Various studies have shown that plumbagin is a potent inducer of ROS. The mechanism underlying ROS induction by plumbagin has predominantly been attributed to inhibition of the antioxidant enzymes TrxR [46,47] and glutathione reductase [46]. In addition to TrxR inhibition, Hwang et al. clarified that plumbagin is a substrate of TrxR; it is reduced by TrxR, which inhibits the interaction of active TrxR with its substrate oxidized thioredoxin [46]. Downstream of ROS induction, plumbagin induces mitochondria-dependent apoptosis in hepatocellular cancer [46], lung cancer [46,48], and cervical cancer [46,49,50], leukemia [47], pancreatic cancer [51], oral squamous cell cancer [52,53], and osteosarcoma [54] among others. It also mediates its anticancer effect by inducing ER stress-mediated apoptosis [52,54,55], S/G2 and G2/M cell cycle arrest [48,49,56] and mitochondrial membrane depolarization in an ROS-dependent manner [45]. With respect to signaling pathways, plumbagin was found to inhibit the NF-κB [57], PI3K/AKT/mTOR [58] and MKP1/2 [59] pathways in non-small cell lung cancer, bladder cancer, and lymphoma, respectively. Additionally, plumbagin has been studied as an adjuvant drug to improve the efficacy of existing chemotherapeutic strategies. Namely, plumbagin was reported to improve the efficacy of chemical-based androgen deprivation therapy for prostate cancer in vivo [60] and a synthetic version of plumbagin, PCUR-101, is presently being explored for the same in a Phase I clinical trial (NCT04677855) with patients with metastatic castration-resistant prostate cancer. It was also reported to improve the efficacy of cisplatin in oral squamous cell carcinoma [53]. Collectively, these findings highlight the potential of plumbagin as a pro-oxidative anticancer agent that warrants further research.

#### 2.1.5. Capsaicin 

Capsaicin (trans-8-methyl-N-vanillyl-6-nonenamide) is an alkaloid that is found naturally in plants of the genus *Capsicum* (chili peppers) and contributes to the burning sensation attributed to spice. Various biological properties have been attributed to capsaicin, including anti-inflammatory and analgesic activities. Intriguingly, although a link between chili pepper consumption and oral and gastrointestinal cancers has long been suggested, capsaicin has been reported as both a chemopreventive and as an anticancer agent [61,62]. Capsaicin has been reported to induce ROS-dependent cell death in various cancers, including colorectal [63], prostate [64,65], bladder [66,67,68], and pancreatic [69,70] cancers. It has also been reported to inhibit tumor growth in vivo in mouse xenograft models of prostate [64] and bladder [66] cancers. Mechanistically, capsaicin-mediated ROS accumulation leads to mitochondrial membrane depolarization [63,64,66], which further triggers mitochondria-dependent apoptosis, as well as G0/G1 cell cycle arrest [68]. Additionally, Sánchez et al. reported that in bladder cancer cells, capsaicin induces JNK activation in an ROS-dependent manner, which results in ceramide accumulation and contributes to apoptosis [65]. Several mechanisms underlying capsaicin-mediated ROS accumulation have been reported, including: (1) inhibition of the activity of antioxidant enzymes SOD, catalase (CAT), and glutathione peroxidase [70]; (2) inhibition of the activity of mitochondrial complex-I and complex-III in the electron transport chain [70]; (3) downregulation of the expression of sirtuin-1, a NAD-dependent deacetylase that regulates the expression of various antioxidant enzymes [69]; (4) upregulation of the expression of NADPH oxidase 4, which generates superoxide [69]; (5) increased expression of FOXO3a, which is a transcription factor that regulates the oxidative stress response [68]. 

#### 2.1.6. Celastrol

Celastrol (24,25,26-trinoroleana-1(10),3,5,7-tetraen-29-oic acid) is a pentacyclic triterpenoid that was isolated from *Tripterygium wilfordii* (thunder duke vine), a plant that is commonly used in traditional Chinese medicine [71]. It has been widely studied as chemopreventive and anticancer drug, and its anticancer activity has been characterized in preclinical models against non-small cell lung [72], breast [73,74], colon [75,76], ovarian [77], gastric [78], and bladder [79] cancers. ROS induction has been attributed as the primary mode through which celastrol mediates its anticancer effects. Downstream of ROS, celastrol has been reported to inhibit HSP90 function [80], induce suppressor of specificity protein (Sp) repressors [79], activate the PKCzeta–AMPK-p53–PLK 2 signaling axis [73], and activate the JNK pathway [80,81] to induce apoptosis. With respect to other ROS-mediated anticancer effects, celastrol induces ER stress [78], mitochondrial dysfunction, specifically disruption of mitochondrial membrane potential [72,78,82], and cell cycle arrest at G2/M phase [76,77] and S phase [75]. Interestingly, at low concentrations (i.e., below the cytotoxic threshold) celastrol was found to induce autophagy in gastric cancer cells through ROS-mediated accumulation of hypoxia-inducible factor 1-α via the transient activation of AKT. However, at cytotoxic concentrations, autophagic flux was inhibited and celastrol mediated p53-independent apoptosis through the JNK pathway [81]. As a pro-oxidative phytochemical, two mechanisms have been reported for ROS induction by celastrol: (1) inhibition of mitochondrial respiratory chain complex I activity [80]; and (2) inhibition of peroxiredoxins, namely peroxiredoxin-1 [76] and peroxiredoxin-2 [78]. The latter is thought to be the main mechanism of action, given that peroxiredoxins are upregulated in many cancer types and are involved in tumorigenesis and chemoresistance [83,84,85]. Consequently, celastrol derivatives are currently in development to improve their potency and specificity against peroxiredoxins [76]. Celastrol has been consistently reported to enhance the sensitivity of triple-negative breast cancer cells to tamoxifen [74] and non-small cell lung cancer cells to erastin [72] and induce ROS-mediated apoptosis in doxorubicin-resistant colorectal cancer cells [75]. These findings highlight the potential of celastrol as an anticancer drug, and its safety is consistently being investigated in an open-label safety study (NCT05494112). However, the clinical translation of celastrol is hindered by poor aqueous solubility, poor bioavailability, as well as potential side effects [71]. Hence, more studies are needed on this front to overcome these limitations to advance the clinical translation of this promising pro-oxidative phytochemical.

### 2.2. Pro-Oxidative Phytochemicals with Uncharacterized Mechanism of ROS Induction

In addition to the above-mentioned phytochemicals, various other phytochemicals have also been investigated for their pro-oxidative capacity with respect to their anticancer activity. However, the mechanism of ROS induction is not well characterized for many of these phytochemicals.

For example, carnosol, a polyphenol, is one such pro-oxidative phytochemical whose mechanism of ROS induction has not been characterized. It was previously identified as an active constituent of sage (*Salvia carnosa*) and rosemary (*Rosmarinus officinalis*) extracts, and its anticancer activity was first characterized in the 1900s [86]. Carnosol was reported to induce ROS-dependent autophagy and/or apoptosis in colon cancer [87], triple-negative breast cancer [88], and osteosarcoma [89] cells. Our lab further clarified the underlying mechanisms of its activity: carnosol was found to target STAT3 [90] and p300 [91] to proteasomal degradation in an ROS-dependent manner and to function as a specific inhibitor of p300 in breast cancer [91]. Additionally, we found that carnosol induced p38-mediated endoplasmic reticulum (ER) stress in an ROS-dependent manner [92], which contributed to carnosol-induced autophagy and apoptosis in triple-negative breast cancer cells. Carnosol was also reported to modulate oxidative stress through depletion of the antioxidant glutathione, which induced apoptosis in adult T-cell leukemia/lymphoma cells [93]. However, the pro-oxidative activity of carnosol appears to be cancer specific, as carnosol has been reported to reduce ROS levels in non-melanoma skin cancer cells [94]. Additionally, very few studies have assessed the anticancer activity of carnosol in vivo, and this is limited to one or two studies each for breast, prostate, and skin cancers [95]. Future research with this promising phytochemical compound should focus on other cancer types and as well as in vivo studies to assess its safety/toxicity and pharmacokinetics. 

Similarly, allicin, a sulfenic acid thioester, which is a major bioactive component of garlic (*Allium sativum*), has been reported to induce cell cycle arrest and apoptosis in an ROS-dependent manner in various cancer cells [96], but the mechanism underlying the observed ROS induction has not been elucidated, to the best of our knowledge. A select few of such pro-oxidative phytochemicals, including allicin, with anticancer activity whose mechanism of ROS induction is not well characterized are listed in Table 1 alongside the anticancer effects induced downstream of ROS induction.

### 2.3. Small Molecules

#### 2.3.1. LCS-1

Lung cancer screen 1 (LCS-1; 4,5-dichloro-2-m-tolylpyridazin-3(2H)-one) was first identified in 2009 from a small molecule screen for lung adenocarcinoma cell lines. It inhibited the growth of *EGFR* or *KRAS* mutant lung adenocarcinoma cell lines by impairing the activation of MAPK and AKT signaling pathways, which regulate cell growth [102]. The same group later identified SOD1 as a protein target for LCS-1 [103]. Consistent with this finding, LCS-1 has been reported to induce its anticancer effect through induction of mitochondrial superoxide and ROS in other cancers, including breast cancer [104], colorectal cancer [105,106], multiple myeloma [107], and glioma [108], which could be attributed to inhibition of SOD1. Downstream of ROS generation, oxidative DNA damage-induced apoptosis [105], ER stress [107], loss of mitochondrial integrity [104], and loss of proteosome function [107] have been reported as anticancer effects of LCS-1. Recently, Ling et al. reported that the anticancer activity of LCS-1 was independent of p53 function and that LCS-1 induced degradation of PARP and BRCA1 [108], suggesting that it inhibited DNA repair pathways. In contrast with numerous in vitro studies, only two studies [107,108] have assessed the in vivo activity of LCS-1 owing to its poor aqueous solubility and, hence, bioavailability. However, LCS-1-loaded triple-polymer-coated magnetite nanocarrier exhibit enhanced efficacy, which could overcome this limitation [109]. Additionally, LCS-1 seems to be a potential therapeutic candidate for bortezomib-resistant multiple myeloma [107] and tamoxifen-resistant breast cancer [110]. Further preclinical research is needed on this SOD1 inhibitor to investigate its anticancer activity in other cancer types, improve its efficacy and pharmacokinetics, and understand the molecular mechanisms underlying its effects.

#### 2.3.2. 15-Deoxy-Δ12,14-prostaglandin J2

15-deoxy-Δ12,14-prostaglandin J2 (15d-PGJ2) is a cyclopentenone prostaglandin, which is a class of biologically active arachidonic-acid derived lipids. 15d-PGJ2 is a well characterized endogenous inhibitor of peroxisome proliferator-activated receptor gamma (PPAR-γ), which regulates inflammation, cell differentiation and glucose mechanism. With respect to its anticancer activity, both PPAR-γ-dependent and -independent mechanisms have been proposed, and ROS induction is one PPAR-γ-independent mechanism of its anticancer activity [111,112]. ROS accumulation-mediated induction of apoptosis by 15d-PGJ2 was first reported in papillary thyroid cancer cells in 2002 [111], and has since been reported in other cancers including glioma [113], prostate cancer [114], colorectal cancer [114,115,116], leukemia [115], non-small cell lung cancer [117], osteosarcoma [118], and breast cancer [119,120]. The following mechanisms have been implicated in the same: activation of JNK in osteosarcoma [118], inactivation of AKT in osteosarcoma and colorectal cancer [115,118], inactivation of the PKA–PLK1 pathway in colorectal cancer [118], and inactivation of the IKKβ–ΝF-κB pathway in prostate cancer [121], and upregulation of DR5 through ROS-mediated induction of CHOP in colorectal cancer [114]. Additionally, 15d-PGJ2 can induce the expression of 15-hydroxyprostaglandin dehydrogenase, which is involved in the inactivation of oncogenic prostaglandin 2, through ROS-mediated activation of ERK1/2 and, subsequently, Elk-1 in breast cancer cells [120]. Interestingly, Cho et al. reported that the MAPK pathway was not involved in ROS-dependent 15d-PGJ2-mediated apoptosis [113]. The induction of ROS by 15d-PGJ2 can be attributed to induction of NADPH oxidase [115] and heme oxygenase-1, which degrades heme, generating ferrous ions that can produce ROS through the Fenton reaction [119,122]. Recently, Na et al. reported that the electrophilic α,β-unsaturated carbonyl group of 15d-PGJ2 is essential for ROS induction as 9,10-dihydro-PGJ2, a non-electrophilic analogue of 15d-PGJ2, failed to induce ROS and ROS-dependent apoptosis [121]. Additionally, 15d-PGJ2 can reportedly modulate the activities of transcription factors involved in redox homeostasis, namely NF-κB, activator protein-1, and Nrf2 [123], which could further account for its ROS-mediated anticancer effects. Although 15d-PGJ2 has shown promise as a pro-oxidative anticancer drug, further research is needed to verify the context-dependent role of this PPAR-γ inhibitor. Specifically, ROS accumulation by 15d-PGJ2 has been reported to stabilize hypoxia-inducible factor-1α, which is a protumorigenic transcription factor, through direct modification of its inhibitor prolyl-4-hydroxylase 2 [119].

#### 2.3.3. Tetraethylthiuram Disulfide

Apart from developing novel anticancer drugs, repurposing drugs that have previously been approved for other conditions can also prove beneficial for the treatment of cancers. Disulfiram (tetraethylthiuram disulfide [DSF], also known as antabuse) is one such drug that is approved by the FDA for the treatment of alcoholism. Its pharmacodynamics and pharmacokinetics are well established, and its commercial availability is widespread. Recently, DSF-Copper (Cu) complexes have been shown to act as potent ROS inducers, specifically through the MAPK/ERK and PI3K/AKT pathways [124]. Cu is known to generate ROS; however, its use in therapeutics is limited because of its tightly controlled intracellular transport. This limitation can reportedly be overcome through complex formation with the DSF derivative N,N-diethyldithiocarbamate, which induces ROS-mediated apoptosis [125]. DSF/Cu complexes have been shown to increase ROS levels and induce G0/G1 cell cycle arrest in acute myeloid leukemia [126], and specifically trigger MAPK-mediated apoptosis in gastric cancer in an ROS-dependent manner [127]. Cu dependent ROS induction through DSF has been used to target prostate cancer, breast cancer, and lymphoid malignancies in preclinical studies [128,129,130]. Apart from Cu, other metal ions have also exhibited anticancer properties and great potential as cancer therapeutics. Namely, gold (III)-dithiocarbamate complexes were shown to induce ROS in breast cancer in part by interfering with the proteosome [131], as ROS can cause cellular disruptions that lead to the inactivation of the ubiquitination–proteasome pathway.

## 3. Pro-Oxidative Drugs in the Clinical Setting

This section discusses pro-oxidative anticancer drugs that are being investigated in clinical trials or are already in clinical use for cancer therapy. Table 2 and Table 3 summarize clinical trials for the drugs discussed in this section.

### 3.1. Pro-Oxidative Drugs in Clinical Trials

#### 3.1.1. Choline Tetrathiomolybdate (ATN-224)

Choline tetrathiomolybdate (ATN-224) is an analogue of the copper chelator tetrathiomolybdate that is used for the treatment of Wilson’s disease. ATN-224 was reported to exhibit anticancer activity via inhibition of SOD1, which resulted in increased superoxide levels and subsequent induction of ROS-dependent apoptosis in multiple myeloma cells [132]. This effect was further characterized in vitro in liver, ovarian, non-small cell lung cancer, colorectal, and pancreatic cancer [2]. Consistently, ATN-244 has been investigated in various clinical trials for solid organ tumors (including prostate, breast, esophageal, colorectal, and hepatocellular cancer) and multiple myeloma (Table 2) [2]. However, the drug has not yet entered any Phase 3 trials. Recently, ATN-224 has been increasingly promoted as an adjuvant drug that can reverse chemoresistance. Combination treatment with cisplatin was found to increase ROS levels, decrease glutathione levels, and increase Platinum–DNA adduct formation, which enhanced the anticancer activity of cisplatin both in vitro and in vivo for non-small cell lung cancer [133]. Ryumon et al. previously reported similar findings for head and neck squamous cell carcinoma [134]. Additionally, the same study reported that pretreatment with ATN-224 sensitized the cisplatin-resistant A431-CDDP-R cell line to cisplatin due to suppression of ATPase copper transporting beta, suggesting that ATN-224 can be used as a chemosensitizing agent. Further studies are needed to assess the potential clinical use or repurposing of ATN-224 as a chemosensitizing adjuvant drug for other chemotherapeutic agents and cancer types.

#### 3.1.2. 2-Methoxyoestradiol

2-methoxyoestradiol (Panzem), an estradiol metabolite, and its formulation, 2-methoxyoestradiol nanocrystal colloidal dispersion, have been investigated in Phase 1 and 2 clinical trials for glioblastoma multiforme, ovarian cancer, multiple myeloma, prostate cancer, and renal cell carcinoma (Table 2), and have FDA orphan drug designation for the former three [135]. The anticancer activity of 2-methoxyoestradiol can be attributed to increased ROS production through a debated mechanism, resulting in a loss of mitochondrial membrane potential [136,137] and nuclear localization of nitric oxide (NO) synthase, resulting in increased NO production and subsequent NO-induced DNA damage [138,139], both of which trigger apoptosis. Ongoing research involving 2-methoxyoestradiol is aimed at combating its main limitation, which is its poor bioavailability. Consistently, sulphamoylated analogues [140,141] and nanomedicine-based approaches [142,143] have been reported to improve the pharmacokinetics of 2-methoxyoestradiol in vivo and in patient-derived xenograft models.

#### 3.1.3. Curcumin and Its Derivatives

Curcumin is the bioactive component of *Curcuma longa* L. (turmeric) and has been actively studied in the past few decades for its various pharmacological properties, including anticancer activity. Over the years, various derivatives of curcumin have been developed to improve its bioavailability and stability [144]. ROS induction has been implicated as one of the mechanisms of the anticancer activity of curcumin and its derivatives in various cancers, including leukemias [145,146], prostate cancer [147,148], colorectal cancer [149,150], gastric cancer [151,152], lung cancer [149,153], glioma [154], breast cancer [149], and cholangiocarcinoma [155]. Curcumin induces ROS by inhibiting the activity of various ROS-related metabolic enzymes, such as CAT, SOD1, glyoxalase 1, and NAD(P)H dehydrogenase [quinone] 1 [146,149]. ROS accumulation further mediates G1 or G2/M cell cycle arrest [146,147,150,154], senescence [146], and apoptosis. Many pathways have been implicated in ROS-mediated induction of apoptosis by curcumin, including downregulation of AKT phosphorylation [145], endoplasmic reticulum stress (namely through the PERK–ATF4–CHOP axis) [150,151,153], activation of the JNK pathway [151], and inhibition of STAT3 [155]. Curcumin has been studied extensively in in vivo cancer models [156] and has been investigated in Phase I and II for various cancers, including multiple myeloma, lung, breast, colorectal, and prostate cancers, with generally beneficial results (Table 2) [23]. Presently, there are two ongoing clinical trials for curcumin for invasive breast (NCT03980509) and unresectable pancreatic (NCT02336087) cancers. Additionally, the combination of curcumin and piperine, a pro-oxidative phytochemical that drastically increases the bioavailability of curcumin in humans [99], is also being investigated in clinical trials (NCT02598726 [Phase 1] and NCT04731844 [Phase 2]). 

### 3.2. Pro-Oxidative Drugs in Clinical Use

Many of the chemotherapeutic drugs that are currently used as standard-of-care treatment for cancers are known to induce ROS. However, the induction of ROS may not necessarily be the main mechanism of action, nor a direct consequence of the drug. For example, paclitaxel, which is used for the treatment of breast, endometrial, and ovarian cancers, exerts its anticancer effect by stabilizing tubulins. This prevents the disassembly of microtubules and consequently induces mitotic arrest, which in turn induces cell death [157]. ROS induction has been suggested as a secondary/alternative mechanism of action of paclitaxel and has been reported in osteosarcoma [158], prostate cancer [159], and non-small-cell lung cancer [160] cells. However, of these studies, only one reported that the use of the ROS scavenger N-acetylcysteine abrogated paclitaxel-mediated effects [159]. Moreover, it has only recently been clarified that mitochondrial accumulation, a consequence of paclitaxel-induced mitotic arrest, causes mitochondrial oxidative stress [161]. This lack of in-depth clarification is true for other approved and clinically used drugs with proposed pro-oxidative activities, such as rituximab, used for the treatment of B-cell lymphomas [10]. Hence, it is important to clarify whether ROS induction directly plays into the anticancer effects mediated by chemotherapeutic drugs.

In this section, we have highlighted cisplatin and doxorubicin as examples of approved/standard-of-care anticancer drugs that directly induce ROS, which further contributes to their anticancer activity. Other examples include motexafin gadolinium (an electron acceptor that increases superoxide production; used in the treatment of breast cancer and malignant melanoma) [162], arsenic trioxide (inhibits SOD and TrxR; used in the treatment of relapsing acute myeloid leukemia) [135], and imexon (disrupts GSH activity, causing depletion of GSH pool; used in the treatment of ovarian cancer, multiforme glioblastoma, and multiple myeloma) [135,162].

#### 3.2.1. Cisplatin

Cisplatin is a platinum-based chemotherapeutic drug that is widely used for the treatment of several cancers, including ovarian, bladder, and testicular cancers. The main mechanism of the anticancer activity of cisplatin can be attributed to DNA–platinum adduct formation, which induces p53-mediated cell cycle arrest and apoptosis [163]. However, oxidative stress independent of nuclear DNA damage has been implicated as one of the mechanisms underlying its cytotoxic effect [164]. Cisplatin induces generation of mitochondrial ROS, which compromise mitochondrial function and membrane potential [165], mitochondrial DNA integrity, and promote mitochondrial biogenesis [164,166], the latter of which further increases mitochondrial ROS levels. Consistently, mitochondrial content was found to correspond to cisplatin sensitivity in ovarian cancer; pharmacological increase in ROS sensitized cisplatin-resistant ovarian cancer cells to cisplatin-induced oxidative stress-mediated apoptosis [166]. Downstream of mitochondrial ROS, cisplatin induces ROS-dependent autophagy and apoptosis through the JNK [167] and Bax/Bak pathways [166], respectively. In contrast to nuclear-DNA damage-mediated cytotoxic effects, cisplatin induces oxidative stress independent of p53 status in head and neck squamous cell carcinoma [168]. The clinical use of cisplatin is often complicated by chemoresistance, as well as associated toxicities, including nephrotoxicity and hepatotoxicity. However, the use of cisplatin in combination with other therapeutic strategies can overcome these challenges. Consistently, there are numerous ongoing clinical trials that are investigating cisplatin in combination with other therapeutic strategies (Table 3).

#### 3.2.2. Doxorubicin

Doxorubicin (Adramycin) is a widely used chemotherapeutic agent for various solid (including breast, ovarian, gastric, and thyroid cancers) and hematological cancers (including multiple myeloma, and acute lymphoblastic/myeloblastic leukemia), either alone, in combination with other drugs (such as cyclophosphamide), or as nanoformulations (such as Doxil) [169]. Although topoisomerase II inhibition is the main mechanism of action, doxorubicin also induces ROS generation through redox cycling into its unstable semiquinone, which releases ROS on conversion back to doxorubicin [170]. Additionally, doxorubicin as an iron chelator forms complexes that catalyze the conversion of H_2_O_2_ and superoxide into hydroxyl radicals or iron–peroxo complexes [169] and downregulates CAT and manganese superoxide dimutase activity [171,172]. Further, ROS induction by doxorubicin mediates p53-independent apoptosis in osteosarcoma cells [172]. Currently, various drug delivery approaches are being investigated to increase the bioavailability of doxorubicin as well as to decrease its side effects, namely cardiotoxicity. Consistently, liposomal and pegylated liposomal doxorubicin are in clinical use for breast cancer [173]. Additionally, pegylated liposomal doxorubicin and other drug delivery approaches for doxorubicin are being studied in clinical trials alone or in combination with other chemotherapeutic agents (Table 3). In this regard, doxorubicin-loaded nanoparticles have also shown promise in in vitro and in vivo studies [174,175,176]. Zheng et al. reported that doxorubicin loaded arginine–glycine–aspartic acid-modified solid lipid nanoparticles exhibited better cellular uptake in both breast cancer and normal cells and higher cytotoxicity against breast cancer cells than doxorubicin in vitro [176]. Additionally, they exhibited higher plasma concentrations and better biodistribution (less drug concentration in kidneys and hearts) compared to doxorubicin in vivo.

## 4. Concluding Remarks

**Figure 2:**
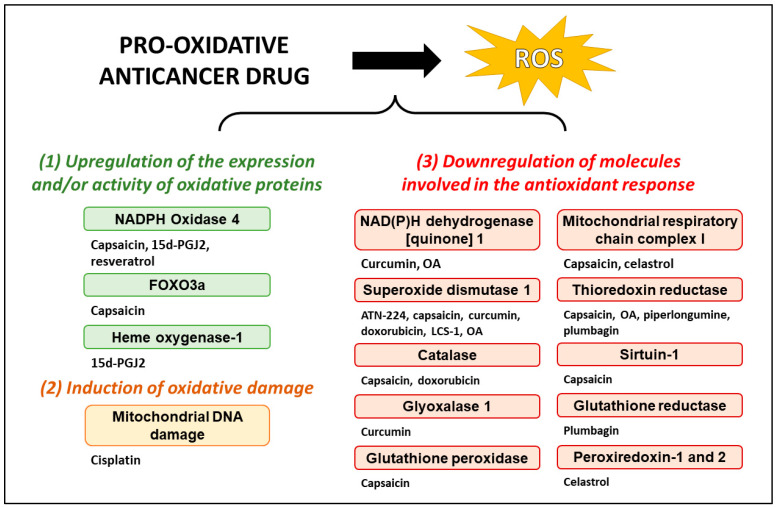
Mechanisms of ROS modulation by pro-oxidative drugs. Anticancer drugs with pro-oxidative function modulate ROS levels through various mechanisms that influence both antioxidant and oxidant molecules involved in maintaining redox balance. The mechanisms utilized by the pro-oxidative anticancer drugs highlighted in this review are summarized in this figure. ATN-224—choline tetrathiomolybdate; LCS-1; Lung cancer screen 1—4,5-dichloro-2-m-tolylpyridazin-3(2H)-one; OA—oleanolic acid; ROS—reactive oxygen species; 15d-PGJ2—15-deoxy-Δ12,14-prostaglandin J2.

**Figure 3:**
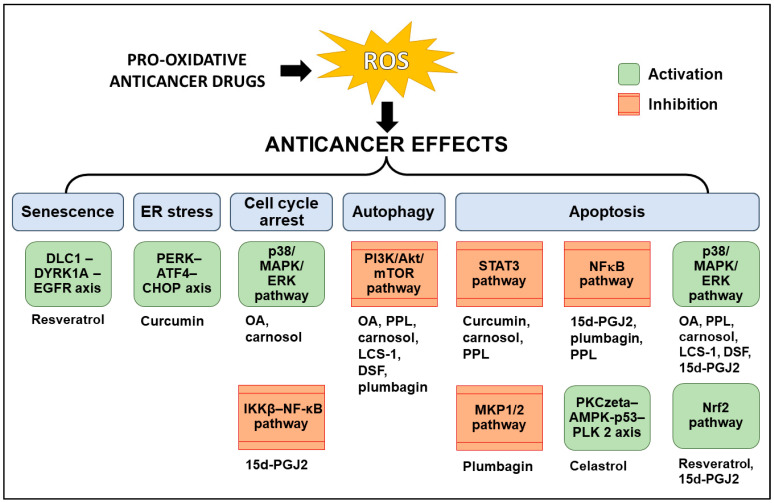
Pathways through which pro-oxidative drugs mediate their anticancer effects in a ROS-dependent manner. Pro-oxidative anticancer drugs mediate their anticancer effects, such as autophagy, apoptosis, cell cycle arrest, ER stress, and senescence, downstream of ROS induction through ROS-dependent activation of various signaling pathways. The signaling pathways activated by the pro-oxidative anticancer drugs highlighted in this review are summarized in this figure. ATN-224—choline tetrathiomolybdate; DSF—disulfiram; ER—endoplasmic reticulum; LCS-1; Lung cancer screen 1—4,5-dichloro-2-m-tolylpyridazin-3(2H)-one; OA—oleanolic acid; PPL—piperlongumine; ROS—reactive oxygen species; 15d-PGJ2—15-deoxy-Δ12,14-prostaglandin J2.

The paradoxical role of ROS in cancer cells presents a unique therapeutic approach that has garnered much attention in recent years. As highlighted in this review, pro-oxidative anticancer drugs, such as cisplatin and doxorubicin, are already being used clinically to treat various cancers, and other pro-oxidative anticancer drugs, such as curcumin and its derivatives, carnosol, and 15d-PGJ2, are in various stages of research and development. The pro-oxidative drugs discussed in the present review induce ROS through diverse mechanisms (Figure 2), which further regulate various signaling pathways to induce anticancer effects (Figure 3); this has been schematically summarized in Figure 2 and Figure 3.

As presented herein, targeting the skewed redox balance of cancer cells via pro-oxidative drugs, specifically pro-oxidative phytochemicals, presents a promising therapeutic approach; however, further research is needed on this front. One of the main aspects which seems to be overlooked is the characterization of mechanisms through which anticancer drugs induce ROS, as reflected with respect to pro-oxidative phytochemicals in Table 1. This is of specific importance as it can help direct the selection of pro-oxidative anticancer drugs by clinicians. For example, drugs that inhibit SOD1 can potentially be used for the treatment of cancers in which SOD1 is overexpressed, such as non-small cell lung cancer and breast cancer [177]. Additionally, although numerous pro-oxidative phytochemicals have been reported to exhibit promising anticancer activity in vitro, there is a lack of sufficient in vivo studies. Moreover, poor bioavailability, a limitation common to many phytochemicals [178], further complicates the matter and hinders their clinical application. Consistently, nanoparticle-based approaches are being investigated to overcome this limitation; however, further research is needed on this front to circumvent the inherent challenges created by this approach, which can limit clinical translation, such as target specificity and toxicity to normal cells [179].

The context-dependent role of phytochemical compounds also needs to be investigated further, as based on the concentration used, phytochemicals, namely resveratrol, are known to function as both antioxidants and pro-oxidants. Although antioxidants have been proposed as adjuvants to counteract the side effects of chemotherapy, the rationale for the same is debatable. While ROS drive cancer cell survival and progression, the disrupted redox balance is further augmented by pro-oxidative anticancer drugs (Figure 1). Given that many of the clinically approved anticancer drugs induce ROS either directly or indirectly, the use of antioxidants for anticancer therapy as opposed to cancer prevention seems counterproductive and could potentially decrease the ROS-dependent anticancer activity of such drugs [3,180]. Hence, it is paramount that in the future, the concentration- and context-dependent roles of phytochemicals should be carefully investigated, with specific focus on the pro-oxidative effects. Moreover, investigation on this front would further facilitate the use of pro-oxidative phytochemicals as adjuvant drugs that can synergize with or sensitize resistant cells to clinically used pro-oxidative anticancer agents; for example, PPL in combination with platinum-based drugs, such as cisplatin [18] and oxaliplatin [19]. Further, it is important to clarify whether ROS induction plays directly into the anticancer effects of chemotherapeutic drugs and to elucidate the underlying mechanisms to exploit the above-mentioned synergistic effects.

## Figures and Tables

**Table 1:** Pro-oxidative phytochemical compounds that exhibit anticancer activity whose mechanism of ROS induction has not been characterized.

Phytochemical	Chemical Class	Plant Source	Cancer Types	Mechanisms Downstream of ROS Induction	Refs
Allicin 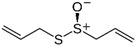	Thiosulfinate	*Allium sativum* (garlic)	Hepatocellular, lung,	Mitochondria-dependent apoptotic cell death	[96]
			Hepatocellular	Mitochondrial membrane depolarization	
			Lung, leukemia	G2/M cell cycle arrest	
Emodin 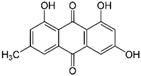	Anthraquinone	*Rheum palmatum* (Chinese rhubarb)	Lung, breast, colon, cervical, prostate, oral squamous cell	Mitochondria-dependent apoptotic cell death	[97]
			Lung, hepatocellular, colon, cervical	Mitochondrial membrane depolarization	
			Hepatocellular, breast, gastric, colon, cervical	G0/G1 cell cycle arrest	
			Gastric, colon, lung	G2/M cell cycle arrest	
			Hepatocellular	↑ Cyclophilin D expression	
			Pancreatic, gall bladder, ovarian	↓ Survivin expression	
			Oral squamous cell	ER stress	
			Cervical, lung, breast, oral squamous cell	Oxidative DNA damage	
Salvicine 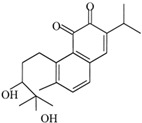	Diterpenoid quinone	*Salvia prionitis*	Leukemia, breast	Oxidative DNA damage	[98]
Piperine 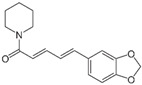	Alkaloid	*Piper nigrum* (black pepper) *Piper longum* (long pepper)	Prostate, cervical, oral squamous cell, rectal, breast, ovarian	Mitochondria-dependent apoptotic cell death	[99,100]
			Oral squamous cell, breast	Mitochondrial membrane depolarization	
			Rectal	G0/G1 cell cycle arrest	
			Oral squamous cell	G2/M cell cycle arrest	
Cryptotanshinone 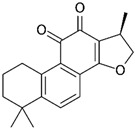	Abietane diterpenoid	*Salvia miltiorrhiza* (Chinese sage) *Salvia przewalskii* (red sage)	Breast	↓ Survivin expression	[101]
			Gastric	Inhibition of AKT pathway	
			Colon	Inhibition of p38–MAPK–NF-κB signaling pathway	
			Gastric	G2/M cell cycle arrest	
			Gastric, leukemia,	Mitochondria-dependent apoptotic cell death	
			Melanoma, lung	↑ Death receptor 5 expression	
			Hepatocellular, lung, breast, lymphoma, gastric, cervical	ER stress	

ER—endoplasmic reticulum; ROS—reactive oxygen species; ↑—upregulation; ↓—downregulation. Chemical structures were taken from PubChem (https://pubchem.ncbi.nlm.nih.gov/), accessed on 27 April 2023.

**Table 2:** Clinical trials investigating pro-oxidative anticancer agents.

Pro-Oxidative Drug	Mechanism of ROS Induction	Cancer	Clinical Trial		
			Phase	ID	Status
Choline tetrathiomolybdate (ATN-224)	Inhibition of superoxide dismutase 1	Breast	II	NCT00674557	Terminated
				NCT00195091	Active, not recruiting
		Prostate	II	NCT00150995	Completed
		Non-small cell lung	I	NCT01837329	Completed
		Lung	I	NCT00560495	Withdrawn
		Multiple myeloma	I/II	NCT00352742	Terminated
		Esophageal	II	NCT00176800	Completed
		Colorectal	II	NCT00176774	Completed
		Hepatocellular	II	NCT00006332	Completed
2-methoxyoestradiol	Unknown	Recurrent glioblastoma multiforme	II	NCT00306618	Completed
				NCT00481455	Completed
		Refractory multiple myeloma	I	NCT00028821	Completed
			II	NCT00592579	Completed
		Prostate	II	NCT00394810	Completed
		Ovarian	II	NCT00400348	Completed
		Unspecified adult solid tumor	I	NCT00030095	Completed
		Carcinoid tumor	II	NCT00328497	Completed
		Metastatic renal cell	II	NCT00444314	Completed
Curcumin *	Inhibition of catalase, superoxide dismutase 1, glyoxalase 1, and NADPH dehydrogenase [quinone] 1	Prostate	I	NCT04403568	Withdrawn
			II	NCT03493997	Completed
			III	NCT02064673	Recruiting
				NCT03769766	Recruiting
		Breast	I	NCT03980509	Active, not recruiting
			II	NCT01042938	Completed
				NCT03072992	Completed
		Colorectal	I	NCT01859858	Completed
				NCT01294072	Recruiting
				NCT01333917	Completed
			II	NCT02439385	Completed
		Pancreatic	I	NCT02336087	Active, not recruiting
			II	NCT00192842	Completed
		Head and neck	I	NCT01160302	Completed
			II	NCT04208334	Completed
		Cervical	I	NCT01035580	Completed
			II	NCT04294836	Withdrawn
		Lung	I/II	NCT01048983	Withdrawn
			II	NCT03598309	Recruiting
			III	NCT04871412	Recruiting
		Leukemia	II	NCT05045443	Recruiting
				NCT02100423	Completed
		Multiple myeloma	II	NCT04731844	Recruiting
				NCT01269203	Withdrawn

*, selected clinical trials are shown for each cancer type. Clinical trial data were taken from ClinicalTrials.gov (https://clinicaltrials.gov/), accessed on 27 April 2023.

**Table 3:** Selected ongoing clinical trials for cisplatin and doxorubicin investigating combination therapies and alternative drug delivery approaches.

Pro-Oxidative Drug	Mechanism of ROS Induction	Cancer	Clinical Trial		
			Intervention	Phase	ID
Cisplatin	Mitochondrial DNA damage	Breast	Cisplatin in combination with gemcitabine	II	NCT04297267
			Cisplatin in combination with veliparib	II	NCT02595905
		Ovarian	Cisplatin in combination with palbociclib	I	NCT02897375
		Lung	Cisplatin in combination with gemcitabine and nadumolimab	I/II	NCT05116891
Doxorubicin	Redox cycling to semiquinone radical Inhibition of catalase and manganese superoxide dismutase	Breast	PLD in combination with and cyclophosphamide	II	NCT01210768
			PLD in combination with IN10018	II	NCT05830539
		Hepatocellular	Doxorubicin-eluting beads	I	NCT05093920
			Doxorubicin in combination with sorafenib	II	NCT01840592
		Ovarian	PLD in combination with pembrolizumab and bevacizumab	I	NCT03596281
			PLD in combination with carboplatin	IV	NCT01210768

PLD—pegylated liposomal doxorubicin. Clinical trial data were taken from ClinicalTrials.gov (https://clinicaltrials.gov/), accessed on 16 May 2023. The status of all the listed trials is active and not recruiting.

## Abbreviations

15d-PGJ2	15-deoxy-Δ12,14-prostaglandin J2.
ATN-224	Choline tetrathiomolybdate.
DSF	Tetraethylthiuram disulfide.
ER	Endoplasmic reticulum.
LCS-1	Lung cancer screen 1.
NO	Nitric oxide.
OA	Oleanolic acid.
PPAR-γ	Peroxisome proliferator-activated receptor gamma.
PPL	Piperlongumine.
ROS	Reactive oxygen species.
SOD	Superoxide dismutase.
TrxR	Thioredoxin reductase.
